# Active rehabilitation for chronic low back pain: Cognitive-behavioral, physical, or both? First direct post-treatment results from a randomized controlled trial [ISRCTN22714229]

**DOI:** 10.1186/1471-2474-7-5

**Published:** 2006-01-20

**Authors:** Rob JEM Smeets, Johan WS Vlaeyen, Alita Hidding, Arnold DM Kester, Geert JMG van der Heijden, Antonia CM van Geel, J André Knottnerus

**Affiliations:** 1 Rehabilitation Centre Blixembosch, Eindhoven, and Netherlands School of Primary Care Research, University of Maastricht, The Netherlands; 2 Department of Medical, Clinical and Experimental Psychology, University of Maastricht, The Netherlands; 3 Department of Education & Research, Atrium Medical Centre, Heerlen, The Netherlands; 4 Department of Methodology and Statistics, University of Maastricht, The Netherlands; 5 Julius Centre for Health Sciences and Primary Care, University Medical Centre Utrecht, The Netherlands; 6 Netherlands School of Primary Care Research, University of Maastricht, The Netherlands

## Abstract

**Background:**

The treatment of non-specific chronic low back pain is often based on three different models regarding the development and maintenance of pain and especially functional limitations: the deconditioning model, the cognitive behavioral model and the biopsychosocial model.

There is evidence that rehabilitation of patients with chronic low back pain is more effective than no treatment, but information is lacking about the differential effectiveness of different kinds of rehabilitation. A direct comparison of a physical, a cognitive-behavioral treatment and a combination of both has never been carried out so far.

**Methods:**

The effectiveness of active physical, cognitive-behavioral and combined treatment for chronic non-specific low back pain compared with a waiting list control group was determined by performing a randomized controlled trial in three rehabilitation centers.

Two hundred and twenty three patients were randomized, using concealed block randomization to one of the following treatments, which they attended three times a week for 10 weeks: Active Physical Treatment (APT), Cognitive-Behavioral Treatment (CBT), Combined Treatment of APT and CBT (CT), or Waiting List (WL). The outcome variables were self-reported functional limitations, patient's main complaints, pain, mood, self-rated treatment effectiveness, treatment satisfaction and physical performance including walking, standing up, reaching forward, stair climbing and lifting. Assessments were carried out by blinded research assistants at baseline and immediately post-treatment. The data were analyzed using the intention-to-treat principle.

**Results:**

For 212 patients, data were available for analysis. After treatment, significant reductions were observed in functional limitations, patient's main complaints and pain intensity for all three active treatments compared to the WL. Also, the self-rated treatment effectiveness and satisfaction appeared to be higher in the three active treatments. Several physical performance tasks improved in APT and CT but not in CBT. No clinically relevant differences were found between the CT and APT, or between CT and CBT.

**Conclusion:**

All three active treatments were effective in comparison to no treatment, but no clinically relevant differences between the combined and the single component treatments were found.

## Background

Chronic non-specific low back pain (CLBP) and the resulting functional limitations have become an epidemic health and socioeconomic problem [1,2]. Many models and therapies have been postulated and applied in order to reduce the burden placed on the individuals with CLBP and society [3-8]. In the literature, three frequently used models regarding the development and maintenance of CLBP functional limitations are described:

1) The physical deconditioning model assuming that loss of muscle strength and endurance including aerobic capacity is responsible for reduced activity levels and hence functional limitations [9,10].

2) The cognitive-behavioral model postulating that functional limitations results from maladaptive beliefs and avoidance behaviors that are maintained by learning processes [11-14].

3) The biopsychosocial model assuming that loss of functional abilities results from both the deconditioning and the cognitive-behavioral model [15].

There is growing evidence that strengthening exercises combined with aerobic exercises as well as cognitive-behavioral treatment (CBT) are worth the effort when compared to no treatment or waiting list control. But there is insufficient evidence for the effectiveness of strengthening and aerobic exercises versus other active therapies [6,16,17], however just recently, Hayden et al. showed that exercise therapy consisting of individually designed programs, including stretching and strengthening, improves pain and function [18]. Furthermore, a recent review showed that there is moderate evidence for the strengthening of deep low back muscles (R Smeets, D Wade, A Hidding, P van Leeuwen, J Vlaeyen, J Knottnerus: The association of physical deconditioning and chronic low back pain: a hypothesis-oriented systematic review. Accepted for publication in Disability and Rehabilitation). Controversy exists regarding the effectiveness of CBT when compared to alternative active treatments [4,6,8]. Multidisciplinary treatment of at least 100 hours, combining exercise therapy, functional restoration and CBT appeared promising in comparison to other non-multidisciplinary treatments, whereas multidisciplinary rehabilitation programs of less than 30 hours failed to prove improvements on several relevant outcome measures. It should be taken into account although, that there is no consensus about the content, intensity and frequency of the different training sessions and the results are based on a relative low number of studies [7].

However, taking a closer look at the studies included in the meta-analyses, it appears that many therapies are not solely based on one of the three models mentioned above. For example in the studies regarding the effectiveness of CBT, exercise therapy was used in order to increase a patient's level of activity while applying the operant learning principles. As a result, it is not clear whether the improvement is reached by the CBT itself, the exercise therapy or the combination of both. Furthermore, many exercise therapies were not of sufficient intensity, frequency and duration to fulfil the physiologic training principles and therefore should not be classified as real reconditioning or strengthening therapies [16,19-22]. On the basis of the current available research, it appears that the evidence for the effectiveness of model-based treatments is still scarce and many programs used several different treatment techniques often based on several models without knowing what treatment elements or combinations are really necessary to reach positive treatment results. Therefore we designed treatments that are exclusively based on the deconditioning model, the cognitive behavioral model and the biopsychosocial model.

The aim of the current study was to compare the effectiveness of a physical treatment (APT), a cognitive-behavioral treatment (CBT) and a combination of both (CT) by means of a randomized controlled trial. It is hypothesized that all active treatments are more effective in reducing functional limitations compared to waiting list controls (WL).

Furthermore, based on the biopsychosocial model it is assumed that the patients with CLBP are physically deconditioned or have to relearn healthy behaviors. Even both problems might be present. So it might be possible that patients only receiving physical treatment will enhance their aerobic capacity, muscle strength and endurance, but there might also be patients who do not resume their normal daily activities because of for example maladaptive beliefs or avoidance behavior. These problems might even hamper successful physical training. Otherwise people receiving cognitive behavioral treatment might be willing to increase their activity level but physical deconditioning might prevent this. By combining both treatments it seems plausible that more people might decrease their level of functional limitations and therefore it is hypothesized that the combination of APT and CBT shows a larger difference than APT or CBT alone.

This paper reports on the immediate post-treatment effects. One-year follow-up results for the three active therapies will be presented later.

## Methods

### Study population

Between April 2002 and December 2004, patients for the first time referred by general practitioners and medical specialists to three outdoor rehabilitation centers in The Netherlands were invited by their consulting rehabilitation physician to participate. Inclusion criteria were: age between 18 and 65 years, non-specific low back pain (CLBP) with or without radiation to leg for more than 3 months resulting in functional limitations (Roland Disability Questionnaire score > 3) [23], ability to walk at least 100 meters without interruption. Exclusion criteria were: vertebral fracture, spinal inflammatory disease, spinal infections or malignancy, current nerve root pathology, spondylolysis or spondylolisthesis, lumbar spondylodesis, medical co-morbidity making intensive exercising impossible (e.g. cardiovascular or metabolic disease), ongoing diagnostic procedures or treatment for their CLBP at the time of referral or a clear treatment preference. Patients were requested to stop other treatments for their low back complaints, except pain medication. The Symptom Checklist (SCL-90) [24] and the Dutch Personality Questionnaire (NPV) [25] were used to check for psychopathology that would hamper individual or group processes [26]. Further exclusion criteria were: not proficient in Dutch, pregnancy and substance abuse that could interfere with the rehabilitation treatment.

To control for expectation bias, patients were told that the study was being performed to compare three currently used treatments for CLBP, of which the exact efficacy had not yet been established and in case they would be randomized to the WL, they would receive a treatment consisting of similar treatment components as in the trial.

The Medical Ethics Committee of the Rehabilitation Foundation Limburg and the Institute for Rehabilitation Research, Hoensbroek, The Netherlands approved the study protocol.

### Randomization

To ensure balance with regard to the number of patients receiving a specific treatment, for each rehabilitation centre clusters of four consecutive patients were randomized using permuted blocks of size eight. For each rehabilitation centre a randomization list was generated by computer under supervision of an independent statistician. Before recruitment of patients the main researcher prepared sealed opaque envelopes for each rehabilitation centre and numbered them sequentially. Furthermore in each rehabilitation centre the randomization list was handed over to the employee who was responsible for planning the treatments. After the start of recruitment of patients, this employee was the only one who had access to the randomization list. Once the research group had recruited four patients in a participating centre, this employee was informed and asked to make arrangements for the first assessment, followed by the start of the allocated treatment within one or two weeks. After the first assessment, the blinded research assistant handed over the sealed envelope to the patient. In order to make sure that the research assistant stayed blinded, the patient was asked not to open the envelope before leaving the building and under no circumstance tell the research what treatment he was allocated to. The participating therapists, research assistants and referring physicians were not aware of this randomization procedure.

### Interventions

The overall goal of the active treatments was to improve functioning (decrease of functional limitations). Emphasis was put on the responsibility of the patient for making plans to keep on being active after the treatment (generalization). Each treatment lasted 10 weeks and started with the explanation of the rationale of that particular treatment. A written summary of the rationale was given to the patients. In order to assure sufficient contrast between the three different treatments and to avoid incorporating possible confounding elements, all therapists were instructed not to discuss general aspects concerning back pain origin, anatomy and ergonomics. In the fourth and tenth week, the rehabilitation physician responsible for the whole treatment, together with the patient evaluated the treatment and checked the generalization plans.

No other interventions than those that were chosen for the APT, CBT or CT took place. In case of acute and severe psychosocial stress or pathology (severe depression, high risk for suicide or personal problems the patient did not wish to discuss during the group treatment), a consultation of a clinical psychologist or social worker was possible. During this consultation the therapist tried to find out what the exact problem was and consecutively, when judged necessary, arranged for professional help outside the rehabilitation centre. All therapists received an extensive training before the start of the trial. They attended refresher courses; two one-day courses during the first year, and one each year in the next two years of the trial. The clinical psychologists and social workers had at least five years of experience in treating CLBP-patients.

#### Active Physical Treatment (APT)

APT was based on the assumption that a reduced aerobic capacity and muscle deconditioning/disuse, especially of the deep lumbar extensor muscles (multifidus muscle) are present [27]. The duration and intensity of APT were chosen according to the physiologic principles of training [19]. The APT consisted of aerobic training, and three dynamic static strengthening exercises.

APT started with half an hour of *aerobic training *on a bicycle; 5-minute warming up, 20 minutes performing at 65 to 80% of the maximum heart rate (HRmax) followed by a 5-minute cooling down. Before training, the VO2 max was calculated based on a slightly modified sub-maximal Åstrand bicycle test [28]. The target HR was calculated using the formula of Karvonen for patients with an aerobic fitness level lower than or equal to the non-trained Dutch population: HRtarget = HRrest + 50% to 60% (HRmax - HRrest) [19,29]. For patients with an aerobic fitness level higher than the non-trained Dutch population the target rate was: HRtarget = HRrest + 55% to 65% (HRmax - HRrest).

During the training the patient judged the perceived exertion by using the Borg scale ranging from 6–20 [30]. When the patient scored above 14, the next HRtarget was lowered, with a score of 14, the middle level of the two calculated HRtargets was chosen, and with a score lower than 14, the upper HRtarget was aimed at [31].

After two and four weeks the percentage in the above-mentioned formula was increased by 5%. From week three on, the patient also had to sprint three times during one minute to achieve a HR calculated by increasing the percentage in the formula with an additional 10%. After the aerobic training the patient stretched the trunk and leg muscles during five minutes. *Three dynamic-static exercises *were performed at 70% of the one-repetition maximum (1-RM), which allowed 15–18 repetitions until muscular fatigue occurred. Each repetition was performed in a standardized and controlled matter allowing two seconds of concentric movement, five seconds of static contraction and two seconds of the eccentric movement. The patient performed three sessions of 15–18 repetitions. The exercises started gradually with in the first week one exercise per session, two exercises in the second week and three in the third week. After five sessions of performing an exercise, the patient performed a test to establish the 70% of the 1-RM again. The three exercises consisted of leg extension while sitting on knees and hands, trunk lifting and lifting both legs while lying prone on a couch. During the exercises assistive weight by a pulley system or extra weight placed on the body of the patient were used depending on the calculated load (70% of 1-RM). The increment of load was based on the performance of the patient and not on the judgment of the therapists. Only when a patient reported change in pain pattern (e.g. radiation to one leg), the rehabilitation physician was asked whether the training had to be adjusted (dynamic instead of dynamic-static, lowering of load or temporary not performing an exercise). After the dynamic-static exercises the patient stretched the trunk and leg muscles again during five minutes. Two physiotherapists guided a maximum of four patients at a time. Each total APT session took 1 3/4 hours and was given three times a week.

#### Cognitive-Behavioral Treatment (CBT)

CBT was based on the assumption that how individuals with chronic pain behave is a resultant of learning, both through environmental contingencies as through information processing [4]. CBT was aimed to help patients to reach their individual daily life goals, to increase their activity level and to modify dysfunctional beliefs. In this trial, CBT consisted of operant behavioral graded activity training [14,32] and problem solving training [33]. During the *graded activity *(GA), the therapist focused on a time contingent gradual increase or pacing of activities being important and relevant for the patient's personal situation. The patient selected three activities that were of the highest importance but compromised by the pain problem. After the establishment of a baseline, the activity tolerance level was calculated and final treatment goals were set. The patient started performing the selected activities following quotas for each day, starting from 70 to 80% of the baseline with gradually increasing activity levels towards the final treatment goals. The patient was instructed only to perform the agreed amount of activity and not perform less or more, even when he felt capable of doing so. During the training sessions the patient performed one or more of the selected activities, but the most important part of the treatment session was the evaluation of the amount of the activities the patient had performed at home. The patient graphically registered in a personal diary his daily performance. The therapists were instructed to discuss these graphs regularly with the patient, while positively reinforcing any progress towards the pre-set goals. In order to create as much contrast as possible with the APT, no physical training element (e.g. muscle strength or aerobic exercises) was incorporated. Graded activity consisted of two introductory group meetings followed by 18 individual sessions guided by a skilled physiotherapist or occupational therapist. The frequency of the sessions gradually decreased from three to one session a week. In total 11 1/2 hours of treatment were given. The partner was invited to attend the first session and a session in the fourth week of treatment.

The *problem solving training *(PST) started with three initial sessions in which the rationale of training and the skill of positive problem orientation were discussed. Sessions four to ten focused on problem definition and formulation, generation of alternatives, decision-making, implementation and evaluation. Patients received a course book with additional information, a summary of each session and homework assignments. The training of the skills and application were the main focus of the training, rather than one specific problem area. Patients were free to select their own personal problem areas. After each session, homework was provided in order to practice the skills in everyday life. A clinical psychologist or social worker, specifically trained to guide this training, provided 10 sessions of 1 1/2 hours to a maximum of four patients at a time.

#### Combined Treatment (CT)

According to the biopsychosocial approach, CT aimed at restoring functional ability through increased fitness, the reinforcement of health behaviors and the modification of dysfunctional beliefs. CT consisted of APT in combination with PST, both in the same frequency and duration as described before. The patient was told that he first had to gain enough aerobic fitness and muscle strength before increasing his activities. The GA was not started until the third week, and began with the selection of the three relevant activities. By the end of the fourth week the final goals and daily quota were set. In total 19 sessions, with a total duration of 11 hours were given.

#### Waiting List (WL)

The patients assigned to the WL were requested to wait 10 weeks after which they were offered a regular individual rehabilitation treatment. During the waiting period, patients were not allowed to participate in diagnostic or therapeutic procedures because of their CLBP.

### Assessment

Assessments (questionnaires and physical performance tasks) were carried out before treatment and immediately after ten weeks of active treatment, and six and twelve months after completion of the treatment. They were supervised and carried out by blinded research assistants who received a special training and who attended regular refresher courses two or three times a year. The WL-patients were only assessed before and after ten weeks of waiting.

### Baseline data

During the pre-treatment assessment data were collected on age, gender, level of education, employment status, duration of complaints and functional limitations, previous low back surgery, previous treatment, level of radiation of pain to leg, traumatic onset of low back pain, fear of injury and movement (Tampa Scale for Kinesiophobia; TSK) [13,34] and physical activity (Baecke Physical Activity Questionnaire; BPAQ) [35,36].

### Primary outcome measure

The level of low back pain associated functional limitations was measured by the Roland Disability Questionnaire (RDQ) which proved to be a valid and reliable instrument in the evaluation of chronic low back pain treatment [23,37,38].

### Secondary outcome measures: questionnaires

1) The severity of the three patient-specific main complaints by using the approach method from Tugwell et al. [39,40]. At baseline the patient selected three activities he performed frequently, which he perceived as important in his daily life, and which LBP made difficult for him. The severities of these main complaints were rated on a 100-mm visual analogue scale (VAS). This is a valid and reliable method with sufficient responsiveness [38,41].

2) Current pain by using a 100-mm VAS for pain at this moment and the Pain Rating Index (total score) of the McGill Pain Questionnaire, a reliable measure of pain intensity ([42,43].

3) Depression measured by the Beck Depression Inventory [44], a reliable, valid and widely used questionnaire [45].

4) Patient's global assessment of overall result measured by a transitional seven-point ordinal scale (1= vastly worsened, 7 = completely recovered) [38,41].

5) Treatment satisfaction measured by using a 100-mm VAS for the overall treatment provided to the patient.

### Secondary outcome measures: physical performance tasks

Six performance tests were selected out of several performance task batteries described in detail by Simmonds [46], Harding [47] and Mayer [48,49]. All seem to have a fairly good validity and reliability in healthy persons and pain patients and most of them also in CLPB patients. The tests include: 1) five-minutes walking (meters), 2) fifty foot walking (seconds), 3) five times sit to stand, performed twice; average time needed to perform a series of five (seconds), 4) loaded forward reaching by holding a stick with a weight of 2.25 or 4.5 kg in front of the body at shoulder height and extend as far as possible (centimetres), 5) one minute stair climbing (number of stairs), 6) PILE-test weight lifting from floor to waist; the patient has to lift a box with a weight four times within 20 seconds from floor up to a 75 cm high table. After each round of four lifting cycles the weight was increased in a standardized way. The test was stopped when the patient could not lift the weight four times within 20 seconds, the HR exceeded 85% of the maximal HR (0,85 × [220 - age]), or the maximal amount of the weight that could be lifted safely (0,6 × body weight) was reached or the research assistant considered the lifting unsafe. The total number of fully completed cycles of lifting was registered. The research assistants were specifically instructed not to encourage the patient to increase his effort. The patient was only asked to perform the tasks as quickly as possible or to walk or reach as far or lift as much as possible.

### Manipulation check

In order to check whether aerobic capacity and problem solving skills were exclusively manipulated in the APT/CT and CBT/CT respectively, the following assessments were carried out:

1) Predicted VO2 max in ml per kg body mass by using a modified Åstrand submaximal bicycle test [50,51]. It is hypothesized that the aerobic training increases the VO2 max.

2) Problem solving skills by using a recently validated short form of the Social Problem Solving Inventory-Revised (SPSI-R) which consists of three scales: rational problem solving (RPS), negative problem orientation (NPO); and impulsive/careless style (IMP) [52]. It is hypothesized that the problem solving training decreases the NPO.

### Treatment compliance and co-interventions

In order to check whether patients were compliant with the allocated treatment, each therapist kept records on the presence during treatment, the amount of exercise (duration, intensity of exercising by monitoring HR during cycling and amount of repetitions and weight displaced during muscle training) and choice of activities and increase in time of these activities for the GA. Also adverse effects and extra appointments with the rehabilitation physician and therapists were registered. Furthermore, cost-diaries were introduced at the first assessment and patients were asked to fill these out during the treatment period. This was an additional way to check for extra appointments at the rehabilitation centre and to check whether patients received additional diagnostic or therapeutic procedures outside the centre during the treatment period.

It was decided that each patient not attending at least 2/3 of all possible treatment sessions for each training element would be classified as having a protocol deviation. Furthermore, during a consensus meeting, two members of the research team, blinded for the allocated treatment examined protocol deviations reported in the cost diaries. Relevant protocol deviations were defined as; visit to chiropractor or physiotherapist performing manipulation, more than one visit to a regular physiotherapist not performing manipulation, visit to general physician or medical specialist other than just for diagnostic questions (e.g. prescription of other pain medication or facet joint injection) or more than two visits to alternative medicine.

### Sample size

A difference of 2.5 points change in score of the primary outcome measure (RDQ) between each of the three active treatment groups and the waiting list group was considered to be clinically relevant [53]. Based on a 2-sided α of 0.05 and a 1-β of 0.90, with a standard deviation (SD) of the RDQ change of 4, a minimum of 220 patients (55 patients per group) needed to be recruited.

### Statistics

To compensate for possibly skewed randomization results, demographic and baseline variables and outcome measures at pre-test were compared between treatment groups. Variables for which differences between groups at baseline were found (P < 0.1) were added to the regression equation as a covariate.

To account for possible dependence of the outcomes within the clusters of four patients who were randomized together, a random intercept term for these patient clusters was included in all models, using multilevel analyses (SPSS mixed linear).

Multiple linear regression analyses were executed in order to test the hypothesis that APT, CBT and CT were more effective than WL, and whether CT showed the strongest effect, as to the outcome measures.

The initial regression model included the following independent variables: pre-treatment measurement of the outcome variable, type of treatment, age, gender, centre of treatment, variables that turned out to be unequally divided between treatment groups despite randomization (covariates), potential prognostic factors and potential effect modifiers such as fear of injury and movement, level of functional limitations, and level of pain intensity. When the interaction variables (treatment × fear of injury/movement, treatment × level of functional limitations, treatment × pain intensity) were non-significant (p > 0.05), they were removed from the model. In case an interaction variable turned out to be significant (p < 0.05), analyses with regard to the concerning outcome variable were repeated within strata. Next, non-significant co-variables (p > 0.05) were eliminated one by one.

Statistical analyses were carried out according to the intention-to-treat principle: all patients, including withdrawals from treatment and patients with poor compliance remained in the treatment group they were randomized to. If data on outcome measures were missing, the baseline-value-carried-forward method was used and a worst-case analysis by imputing the tenth percentile score of the outcome measure at post-treatment of the respondents was performed as well.

In order to check for manipulation, pre-post difference in the calculated VO2 max was tested by means of paired T-tests within each group receiving sufficient aerobic training (APT and CT) and each group receiving no aerobic training (CBT and WL), and by an independent T-test for the difference in VO2 max between these two groups. The same was done for the groups receiving a sufficient number of problem solving skills training sessions (CBT and CT) and those receiving no such training at all (APT and WL) by testing the differences on the SPSI-NPO subscale.

SPSS statistical software, version 12.0 was used for the statistical analyses (SPSS, Inc., Chicago).

## Results

### Study population

**Figure 1:**
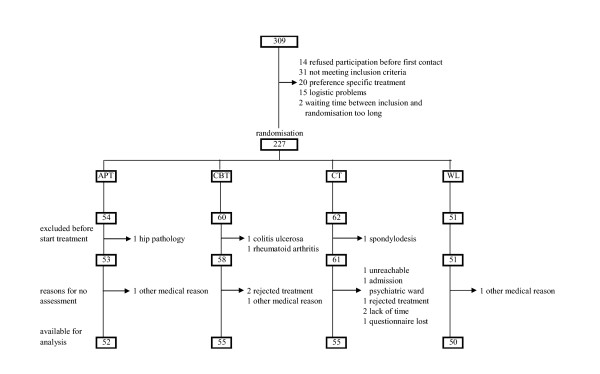
Flow diagram summarizing the formation of the study group and the number of lost to follow-up. APT = active physical treatment, CBT = cognitive behavioral treatment, CT = combination treatment, WL = waiting list.

Of the 309 eligible patients, 82 patients were not included. The reasons for not including these patients are shown in figure 1.

At the start of treatment, four patients, who were randomized to APT (n = 1), CBT (n = 2) and CT (n = 1) respectively, appeared not to fulfil the selection criteria (see figure 1). Because these medical problems already existed before the treatment started, these patients were excluded from further analysis.

Furthermore three patients, although they had a RDQ-score of >3 at the time of inclusion appeared to have a score of ≤ 3 at pre-treatment assessment. One patient completed the APT, another patient stopped the CT after nine sessions because he had only minor functional limitations and could not combine his job with the intensity of treatment. The third patient only attended one session of CBT and stopped further treatment because her complaints were almost completely resolved. These three patients were all included in the intention-to-treat analysis.

Another three patients (one in APT, one in CBT and one in CT) never showed up for treatment but all attended the follow-up assessment and were also included in the intention to treat analysis.

For eleven patients (APT: n = 1, CBT: n = 3, CT: n = 6, WL: n = 1) no data of questionnaires at post-treatment were available (5%). Ten of these patients dropped out of treatment and the reasons for not filling out questionnaires are shown in figure 1. The questionnaire of one patient who completed the treatment got lost in the mail.

For the performance tasks the data were complete for 200 of the 223 patients (90%). The data were missing for five patients in APT, ten in CBT, six in CT and two in WL; one patient was unreachable (CT), five had other non-LBP associated medical or psychological problems (one in APT, CBT, WL and two in CT), ten rejected treatment (two in CT, three in APT and five in CBT), five reported logistic problems (one in CT and WL, three in CBT), one had increase of pain (APT) and one had no complaints anymore (CBT). So only a small percentage of data was missing and the reasons for not responding were not related to the content of treatment (e.g. adverse effects). Nevertheless, a worst-case analysis was performed by imputing the missing data by the tenth percentile score of the available data.

### Comparability at baseline

Demographic variables had similar distribution in the treatment groups (see table 1). Regarding disease characteristics only the duration of functional limitations was not similar distributed in the treatment groups, and was entered into the regression analysis as a covariate. Regarding the outcome measures, the WL performed significantly better at baseline on the forward reach test than the three active treatments. The total group had moderate to severe functional limitations and quite a high percentage of the patients was on sick leave or disability pension because of their low back pain and most of them (97%) already had undergone some sort of physical or medical treatment for their CLBP.

There were no statistically significant differences regarding the baseline variables between the responders and non-responders on the questionnaires. The non-responders on the performance tasks had a significantly lower baseline level of sport activity, fast walking and stair climbing.

### Treatment compliance and co-interventions

In the APT the mean number of sessions for the total group of randomized patients was 24.5 (maximum of 30) for aerobic and strengthening training. In the CBT the mean number of sessions for the GA was 14.3 (maximum of 20) and 7.7 (maximum of 10) for the PST. The CT-patients attended a mean of 21.8 physical training sessions, 11.9 (maximum of 19) GA-sessions and 7.1 PST-sessions.

In APT, 83% of all patients met the criterion of at least 2/3 attendance. Of all CBT-patients, 78% and 76% had a sufficient number of sessions of GA and PST respectively. For the CT-patients, 72% had sufficient physical training and 62% sufficient GA and PST. The main reasons for not completing a sufficient number of treatments were rejection of treatment (n = 23; 4 in APT, 8 in CBT and 11 in CT) or other non-LBP associated medical or psychological problems (n = 11; 3 in APT, 1 in CBT, 7 in CT). For only six patients (three in APT and three in CT), increase of pain was the reason to stop the treatment (see adverse effects). Logistic reasons were only reported by patients attending the CBT or CT (n = 12; 6 in each treatment), mainly because the time of treatment changed regularly or the higher frequency of different training elements and the resulting longer total treatment time. One patient (CBT) quitted treatment because he had no complaints anymore.

Analysis of the cost-diaries that had been returned by 209 patients (94%; APT, n = 51; CBT, n = 52; CT, n = 56; WL, n= 50) showed that an equal number of patients did not completely adhere to the treatment protocol. There were no statistically significant differences between groups regarding the percentage of patients visiting the general physician, medical specialist, radiologist, physiotherapist or alternative medicine and the mean number of visits during the treatment period (table 2). Relevant protocol deviations as defined earlier were reported in 27 patients (5 in APT, 8 in CBT, 4 in CT and 10 in WL).

### Adverse effects and other co-morbidity

Three patients in the APT and three in the CT stopped the treatment because of increased pain in the lower back or radiating leg pain. One of these patients, attending the APT developed three days after a training session a herniated disc with neurological deficits needing neurosurgical intervention. Furthermore, one patient attending the APT stopped the aerobic training because of knee complaints. One patient attending the APT developed pain complaints in both legs during cycling which appeared to be caused by vascular problems that could be resolved by vascular surgery.

### Outcome measures

The observed change on the RDQ, the primary outcome measure without any correction was +0.04 ± 2.90 for the WL, -2.25 ± 4.51 for APT, -2.65 ± 4.66 for CBT and -2.27 ± 4.19 for CT, respectively. In table 3 the results of the multiple linear regression analyses with correction for dependence within patient clusters, gender, age, centre of treatment, duration of functional limitations, and significant prognostic variables are presented. First the mean scores of the WL at post-treatment are shown and next, the mean difference of the three active therapies compared with the score of the WL and the 95% confidence intervals (CI) are presented. The presented differences represent the real difference between the active treatments and WL after correction for any time-effects in the WL. All active treatments showed significant improvement on functional limitations, main complaints and pain intensity measured by using the VAS. The CBT and CT significantly improved on the global improvement measure and the APT almost reached significance. No difference was found on the total score of the Pain Rating Index and only APT showed a significant reduction of depression. Because the outcome measures fast walking and sit-to-stand were not normally distributed at baseline, the inverse of these measures was used. Compared to the WL, both APT and CT showed significant improvements on walking, sit to stand, and stair climbing. Lifting improved significantly in APT and fast walking in CBT and CT.

In table 4 the results of the comparison of CT versus APT and CBT are presented. No differences were found, except that the BDI was significantly more decreased in APT and walking improved significantly more in CT than CBT.

Since the treatment effect for overall satisfaction was modified by the baseline RDQ (interaction), the satisfaction scores for three different percentiles of the baseline RDQ are presented. It appeared that the level of satisfaction was significantly higher in APT compared to WL when the patient had a lower level of functional limitations at pre-treatment. For the ninetieth percentile score (RDQ = 19) this difference was not significant. CBT and CT showed a significantly higher level of satisfaction compared to WL, and the higher the baseline RDQ-score, the greater this difference became. Only for the ninetieth percentile score, CT showed significantly greater satisfaction than APT. No differences were evident between CT and CBT.

The alternative analyses that replaced the missing data according to the baseline-value-carried-forward method showed very similar results, except that the differences on the lifting task and sit-to-stand task were no longer significant for the APT-versus WL-group. The worst-case analysis did not show great differences either. The mean difference between CT and WL regarding current pain was no longer significant (-5.852 [95% CI: -14.192 to 2.489]) as well as the global improvement (0.495 [95% CI: -0.29 to 1.018]). For the performance task a few changes occurred: in comparison to the WL, the sit to stand task was no longer significant in APT and CT, the fast walking task was no longer significant in CBT and CT and the stair-climbing task just did not reach significance anymore in APT.

The multilevel regression analyses showed that the dependence within patient clusters was usually small, intraclass correlations (ICCs) being never larger than 0.15 with only two exceptions; the regression model on loaded forward reach showed an ICC of 0.17 and stair climbing 0.20.

### Manipulation check

The patients who received a sufficient number of aerobic training showed a significant VO2 max improvement of 4.84 mlkg^-1^min^-1 ^versus a non-significant improvement of 0.29 mlkg^-1^min^-1 ^in the CBT and WL-group. The mean difference of 4.55 mlkg^-1^min^-1 ^(95% CI: -2.88 to -6.21 mLkg^-1^min^-1^) was highly significant indicating that the aerobic training was highly effective in increasing the VO2 max in patients receiving a sufficient number of aerobic training sessions.

Both patients who received a sufficient number of PST and those who did not receive this training at all, showed a significant change in the SPSI-NPO score (-1.80 and -1.92 respectively) indicating that the negative problem orientation decreased. But the mean difference of -0.1 (95% CI: -1.18 to 1.43) was not significant, suggesting that the PST had no additional effect in decreasing the SPSI-NPO score.

## Discussion

Although our patients had moderate limitations and most of them were already treated previously, all theory-based treatments, as hypothesized, were more effective than WL. The uncorrected data already showed a relevant decrease of the RDQ-score for all active treatments. After correction for non-balance regarding baseline variables and adjustment for patient cluster dependence and centre of treatment, CBT and CT even showed a clinically relevant decrease of ≥ 2.5 points on the RDQ and APT just did not reach this clinically relevant level (2.4 points). Furthermore, hardly any adverse effects were reported. Several secondary outcomes such as main complaints, current pain and global improvement further confirmed this conclusion. The more the patients had functional limitations the higher they reported treatment satisfaction after attending CBT or CT. Patients attending APT were more satisfied than WL-patients although this difference turned non-significant when pre-treatment functional limitations were high. This indicates that based on the patient's overall satisfaction, administration of APT in patients with moderate to severe functional limitations was less effective.

The performance tasks that seemed to be more physically demanding, improved significantly in treatments with a physical modality.

While comparing CT with APT or CBT, CT showed a significantly higher walking distance than CBT, but it can be debated whether a difference of 22.8 on 419 meters is clinically relevant. APT showed a greater reduction of depression than CT, although this change was not clinically relevant since the mean baseline score of the BDI was already low in all treatment groups. Regarding all other outcome measures, CT was not more effective than APT or CBT respectively, so the hypothesis that CT has a stronger effect than APT and CBT, could not be supported.

Until now, it was not known whether changes in functional limitations are more effectively reduced by purely psychological or physical interventions [3,54]. To our knowledge the present study is the first trial in CLBP comparing explicitly theory-based treatments to WL and CT to APT and CBT respectively, trying to address these problems. Both purely physical and psychological treatments as well as CT showed clinically relevant improvement. The overall results of all active treatment groups are comparable to the results presented in the most recent reviews and meta-analysis on different active treatments [7,8,17,18].

The lack of great differential effects of different active treatments has been attributed to the fact that these treatments are not sufficiently theory-driven [55]. But even in our study, CT showed no additional effect. This might be caused by the relative low compliance rate in CT. Furthermore the total treatment intensity might have been a crucial factor for obtaining an additional effect. Although the CT had a total duration of 78 hours, this is still lower than the 100 hours of therapy in daily intensive CT-programs showing an additional effect when compared to non-multidisciplinary treatment [7]. Furthermore no functional restoration was applied. On the other hand, we cannot rule out that the mixture of APT and CBT had an oppositional effect. For example, the increase of exercise load in APT was based on training physiology, and the increase of activity in CBT was based on time-contingency, which could have obscured the supplemental effect of both treatments.

By using the WL as reference treatment it cannot be ruled out that the positive effects of all active treatments were caused by other non-specific factors such as attention, a standardized treatment program, or emphasis on active participation. Otherwise, by using the WL we were able to control for time effects showing that the WL did not improve or deteriorate on all outcome measures. Furthermore, due to ethical regulations, it was not possible to use an attention-control/placebo treatment once a patient had been given an indication for rehabilitation treatment.

By including a total number of 223 patients and having 212 patients available for analyses, sufficient power was assured for the comparison between the three active treatments and the WL regarding the primary outcome measure, functional limitations. Otherwise, the power might have been insufficient to find differences between the active treatments although the point-estimates showed no clear tendency in favour of the CT.

Although the patient compliance was not very high, 95% of all randomized patients were assessed. This means that most patients, also those who did not have a sufficient intensity or showed serious protocol deviations, were included in the analyses. In this way the intention-to-treat method was approached as much as possible, ensuring that the real effectiveness of theory-based treatments in comparison to WL was determined. The results appeared to be quite robust since the alternative analyses hardly changed our results.

At randomization, all groups were quite similar on demographics and patient characteristics. Only the duration of functional limitations was definitely not equally distributed, but this variable was additionally controlled for in the statistical analyses.

In this study quite liberal inclusion criteria were used. In other studies, for example patients with psychosocial problems were excluded [56] were treated as inpatients and should not have had previous back surgery, ongoing somatic or psychiatric disease or generalized disc degeneration [57]. The level of functional limitations of our patients was relative high compared to that reported in other studies [58-61], but in accordance with the Dutch health care system in which CLBP-patients with moderate to severe functional limitations are treated in outpatient rehabilitation centers [62]. This means that the generalizability of the results for clinical practice is very high.

To improve the quality of the interventions, all treatments were highly structured by using detailed treatment protocols, and given by well-trained and skilled therapists. In order to avoid possible confounding within all treatment elements, therapists were trained only to deliver one specific treatment element in the single and combination treatment as well. Therefore it was not possible to keep the therapists blinded. The patients could not be blinded because of ethical reasons. However, before randomization the patients were told that all active treatments are effective, but that the exact effectiveness is not yet clear. Furthermore, patients with an absolute preference for one treatment were excluded. Concealment of randomization was successfully achieved since no one of the referring physicians was aware of the type of treatment the referred patient would be randomized to. The blinding of the research assistants also seemed to be successfully maintained.

The compliance with treatment protocol by patients and clinicians are rarely assessed or adequately reported in RCTs [63]. Therefore additional registration forms for the therapists and diaries for the patients were used. Inspection of these forms indicated that there were no statistical significant differences between all treatment groups regarding protocol deviations. The treatment quality was judged to be sufficient in those patients who received at least 2/3 of all possible treatment sessions. This was further confirmed by the significant increase in VO2 max in the treatments using a physical training modality. The lack of additional improvement in negative problem solving after completion of PST was also reported by van den Hout et al. [33]. Despite this lack of improvement, van den Hout et al. found a decreased level of functional limitations in the problem solving group at 12-month follow-up, meaning that PST probably exerted its effect otherwise.

Furthermore in only a limited number of patients the reasons for insufficient adherence were exclusively related to the type of treatment. The reported rate of compliance was quite similar to a few other RCTs using comparable treatments, ranging from 68% to 73% [60,64] and even only 69 % in regular multidisciplinary rehabilitation programs [65].

Several investigators recommend that besides subjective questionnaires, more objective outcome measures such as performance tasks should be used [15,46]. The improvement on performance tasks mainly occurred in APT and CT. This might be due to an increase of aerobic capacity or endurance strength. Otherwise, several patients did not report difficulties in, for example stair climbing, and for them a ceiling effect for this particular task seemed to apply. Furthermore, the performance tasks were possibly not specific enough to detect a change in the ability to perform patient relevant activities. For instance when a patient wanted to improve his walking distance, he would have increased the distance and not the speed. Because of this, the five-minute walking task probably would not change dramatically. Another explanation can be that the subjective experience of functional limitations and main complaints, as rated by questionnaires changes more easily, while changes in performance tasks take more time to establish [66]. It is not known what these results mean for clinical practice, since besides our own study, only one RCT using similar performance tasks was identified and the only conclusion was that the number of patients showing improvement on these performance tasks was lower than for the self-rated outcome measures [66].

Since all active treatment groups showed similar effectiveness, the question arises how the effects are mediated. Previous studies have shown that irrespective of the treatment modality, improvement was mediated by the reduction of pain catastrophizing and the increase in experienced pain control [67-69]. Also in our study, patients in all treatment conditions have been exposed to situations that may have challenged their catastrophic beliefs that pain is a serious threat to their health. Results of such a mediation analysis based on our data will be reported later.

In order to improve the effectiveness it is not only necessary to find out how the treatment exerts its effect but also the question "what works for whom?" A way to further explore this might be to look for subgroups of patients by using objective, valid and reliable criteria to enhance the effectiveness of treatment programs [70].

## Conclusion

The results showed that the three theory-based treatments were more effective than WL. However, CT did not show greater differences than APT and CBT respectively. These findings show that APT and CBT are as effective in reducing the personal experienced level of functioning as CT. Given the treatment intensity one could prefer the less intensive treatments instead of the more intensive and therefore more expensive CT. However, one can only decide on cost-effectiveness by comparing APT/CBT with CT after a longer period of follow-up with a proper cost-effectiveness analysis taking the total health care and work-related cost into account. This analysis will be carried out and presented once the one-year follow-up will be completed. Based on the patients' overall satisfaction, CBT is to be preferred when the patient has moderately to severely functionally limitations.

Further research on theory-based treatments to confirm our findings, to investigate mediation and to develop more effective treatments is warranted.

## Competing interests

The author(s) declare that they have no competing interests.

## Authors' contributions

RJEMS: involved in conception and design, obtaining grants, development of interventions and assessment protocol, training of therapists for the physical treatment and graded activity, training of research assistants performing the assessment, performance of trial, acquisition, statistical analyses and interpretation of data, drafting of the article.

JWSV: involved in conception and design, made contributions in obtaining grants, involved in choosing measurements, development of cognitive-behavioral intervention, participation in trial, interpretation of data and substantial contribution in revising the article for important intellectual content.

AH: involved in conception and design, made contributions in obtaining grants, involved in choosing measurements, participation in trial and also partly responsible for coordination, interpretation of data and substantial contribution in revising the article for important intellectual content.

ADMK: participation in trial, very important contribution in the statistical analyses and interpretation of data, and is involved in revising the article for important intellectual content.

GJMGH: involved in conception and design, made contributions in obtaining grants, involved in choosing measurements, participation in trial, interpretation of data and substantial contribution in revising the article for important intellectual content.

ACMG: involved in acquisition, analysis and interpretation of data, partly involved in drafting the article and substantial contribution in revising the article for important intellectual content.

JAK: involved in conception and design, made contributions in obtaining grants, involved in choosing measurements, participation and partly responsible for coordination, statistical analyses and interpretation of data and substantial contribution in revising the article for important intellectual content.

## Pre-publication history

The pre-publication history for this paper can be accessed here:



## Figures and Tables

**Table 1:** Baseline variables for the total population and the four therapy groups (total n = 223)

**Variables**	APT (n = 53)	CBT (n = 58)	CT (n = 61)	WL (n = 51)
Age (years)	42.68 ± 9.06	42.52 ± 9.67	40.67 ± 10.14	40.55 ± 11.17
Gender (% male)	58.5	41.4	62.3	49.0
Education (%)				
low	67.9	62.1	57.4	62.7
middle – high	32.1	37.9	42.6	37.3
Work status (%)				
full time	32.1	36.2	24.6	25.5
partial sick leave /disability pension	18.9	24.1	32.8	17.6
full sick leave/disability pension	43.4	31.0	36.1	43.1
no job/retired	5.7	8.6	6.6	13.7
Duration of LBP (months)	56.91 ± 75.86	68.33 ± 74.21	56.13 ± 67.50	43.75 ± 70.82
Duration of functional limitations (months)	28.85 ± 37.43	49.12 ± 61.40	36.90 ± 53.57	23.55 ± 32.76*
Radiation of pain (%)				
no radiation	9.4	17.2	13.1	15.7
above knee	35.8	27.6	42.6	41.2
below knee	54.7	55.2	44.3	43.1
Previous back surgery (%)	17.0	10.3	14.8	19.6
Trauma preceding LBP (%)	17.0	19.0	11.5	27.5
Sport activity (BPAQ-sport)	2.07 ± 0.67	2.14 ± 0.78	2.04 ± 0.65	2.10 ± 0.67
Leisure activity (BPAQ-leisure)	2.96 ± 0.69	2.79 ± 0.74	2.84 ± 0.60	2.78 ± 0.67
TSK	39.02 ± 6.52	38.72 ± 6.88	39.69 ± 7.08	37.75 ± 6.97
VO2 max (ml/kg/min)	27.26 ± 6.76	28.23 ± 6.69	30.06 ± 7.88	29.32 ± 7.07
SPSI-NPO	13.37 ± 7.42	13.90 ± 6.29	13.34 ± 7.71	15.10 ± 7.66
RDQ	14.15 ± 3.70	13.74 ± 3.65	13.51 ± 3.92	13.96 ± 3.88
Main complaints	74.52 ± 14.59	74.71 ± 16.19	72.44 ± 17.03	77.42 ± 11.35
Current pain	51.23 ± 26.55	48.84 ± 23.51	45.98 ± 23.95	51.02 ± 25.40
PRI-T	18.34 ± 11.32	17.86 ± 9.94	18.08 ± 9.04	17.37 ± 8.52
BDI	10.38 ± 7.62	10.45 ± 7.06	9.75 ± 6.68	9.78 ± 7.67
Walking (m)	375.37 ± 68.03	385.29 ± 75.36	372.40 ± 88.03	383.48 ± 101.75
Fast walking (sec)	9.92 ± 2.27	10.28 ± 2.40	10.31 ± 2.51	10.02 ± 2.80
Sit to stand (sec)	20.98 ± 7.33	22.62 ± 9.99	21.86 ± 7.65	21.19 ± 8.98
Loaded forward reach (cm)	52.04 ± 12.27*	50.12 ± 14.97*	55.00 ± 11.24*	59.62 ± 13.55*
Stair climbing (number of stairs)	72.02 ± 18.22	75.10 ± 24.76	71.26 ± 21.95	78.35 ± 26.90
Lifting (stages)	4.31 ± 2.58	4.29 ± 3.22	4.16 ± 3.05	4.06 ± 2.64

Values presented as means and standard deviation or percentage

* significant differences between therapy groups (p < 0.05)

APT = active physical treatment; CBT = cognitive-behavioural treatment; CT = combination treatment; WL = waiting list; LBP = low back pain; BPAQ = Baecke Physical Activity Questionnaire; TSK = Tampa Scale for Kinesiophobia; SPSI-NPO = Social Inventory Problem Solving – Negative Problem Orientation; RDQ = Roland Disability Questionnaire; PRI-T = Pain Raiting Index Total score; BDI = Beck Depression Inventory.

**Table 2:** Protocol deviations; total percentage and mean number of visits

	APT (n = 51)		CBT (n = 52)		CT (n = 56)		WL (n = 50)	
	%	Mean	%	Mean	%	Mean	%	Mean

General physician	23.5	0.37	25.0	0.81	26.8	0.39	24.0	0.44
Medical specialist	7.8	0.12	7.7	0.15	8.9	0.11	8.0	0.08
Radiologist	0	0	0	0	0	0	0	0
Physiotherapy	11.8	0.53	13.5	0.77	5.4	0.39	18.0	0.90
Alternative medicine	0	0	3.8	0.23	5.4	0.21	4.0	0.16

APT = active physical treatment; CBT = cognitive-behavioural treatment; CT = combination treatment; WL = waiting list.

**Table 3:** Effects of APT, CBT and CT as compared with WL

**Dependent variable**	**WL mean ± SD**	**APT mean difference (95% CI)†**	**CBT mean difference (95% CI)†**	**CT mean difference (95% CI)†**
RDQ††	13.88 ± 4.78	-2.40 (-4.14 to -0.65)**	-3.05 (-4.80 to -1.30)**	-2.56 (-4.27 to -0.85)**
Main complaints††	74.25 ± 14.7	-11.19 (-20.07 to -2.31)*	-16.36 (-25.13 to -7.60)**	-17.84 (-26.54 to -9.14)**
Current pain††	53.35 ± 22.6	-8.68 (-16.87 to -0.48)*	-14.76 (-23.00 to -6.52)**	-8.23 (-16.37 to -0.10)*
PRI-T††	17.28 ± 10.48	0.90 (-2.94 to 4.74)	-2.04 (-5.91 to 1.83)	-0.33 (-4.14 to 3.48)
BDI	9.42 ± 7.81	-2.09 (-3.86 to -0.32)*	-1.65 (-3.42 to 0.12)	0.04 (-1.71 to 1.79)
Global improvement††	3.78 ± 0.91	0.47 (-0.08 to 1.09)	0.90 (0.36 to 1.44)**	0.70 (0.17 to 1.24)*
Satisfaction$				
10th percentile of baseline RDQ (= 9)	45.65 ± 25.30	32.38 (14.19 to 50.57)**	18.34 (0.51 to 36.16)*	19.33 (2.01 to 36.65)*
50th percentile of baseline RDQ (= 14)	46.67 ± 25.30	19.30 (6.74 to 31.88)**	22.68 (10.10 to 35.27)**	23.57 (11.28 to 35.86)**
90th percentile of baseline RDQ (= 19)	47.69 ± 25.30	6.23 (-12.04 to 24.50)	27.03 (8.37 to 45.69)**	27.81 (9.54 to 46.08)**
Walking (m)††	386.60 ± 86.62	27.85 (5.35 to 50.34)*	12.82 (-9.75 to 35.40)	35.65 (13.85 to 57.45)**
Inversion fast walking (1/sec)	0.10 ± 0.02	0.005 (-0.002 to 0.012)	0.009 (0.002 to 0.016)*	0.008 (0.001 to 0.015)*
Inversion sit to stand (1/sec)	0.06 ± 0.02	0.007 (0.001 to 0.013)*	0.003 (-0.003 to 0.010)	0.007 (0.001 to 0.014)*
Loaded forward reach (cm)††	59.07 ± 12.55	1.30 (-4.40 to 7.00)	1.19 (-4.58 to 6.96)	0.98 (-4.54 to 6.51)
Stair climbing (stairs)	80.76 ± 24.80	7.83 (0.67 to 14.98)*	3.58 (-3.53 to 10.69)	9.25 (2.28 to 16.21)*
Lifting (stages)††	4.13 ± 2.56	0.96 (0.06 to 1.85)*	0.48 (-0.41 to 1.37)	0.68 (-0.16 to 1.52)

† Values are the mean difference between this group and the WL. The WL score is the score at post-treatment.

The mean differences and confidence intervals (CIs) and corresponding p-values were estimated adjusting for age, gender, center of treatment, baseline score of outcome measure, duration of functional limitations.

†† Additional correction for relevant prognostic co-variates: RDQ – work status and TSK; Main complaints/Lifting – TSK; Current pain/PRI-T – work status; Global improvement/loaded forward reach – current pain; Walking – radiation and TSK.

$ Data presented for different strata for baseline RDQ, mean and SD for WL are estimated by use of regression model. * p < 0.05; ** p < 0.01.

APT = active physical treatment; CBT = cognitive-behavioural treatment; CT = combination treatment; WL = waiting list; RDQ = Roland Disability Questionnaire; PRI-T = Pain Rating Index Total score; BDI = Beck Depression Inventory.

**Table 4:** Effects of APT and CBT as compared with CT

**Dependent variable**	**CT mean ± SD**	**APT mean difference (95% CI)†**	**CBT mean difference (95% CI)†**
RDQ††	11.40 ± 5.25	0.16 (-1.52 to 1.85)	-0.49 (-2.17 to 1.19)
Main complaints††	54.68 ± 21.79	6.65 (-1.96 to 15.26)	1.48 (-7.04 to 9.99)
Current pain††	42.31 ± 25.56	-0.45 (-8.41 to 7.52)	-6.53 (-14.48 to 1.43)
PRI-T††	17.53 ± 10.53	1.23 (-2.51 to 4.97)	-1.71 (-5.45 to 2.03)
BDI	9.07 ± 6.53	-2.13 (-3.84 to -0.42)*	-1.69 (-3.41 to 0.03)
Global improvement††	4.53 ± 1.33	-0.23 (-0.77 to 0.30)	0.20 (-0.33 to 0.73)
Satisfaction$			
10th percentile of baseline RDQ (= 9)	64.98 ± 25.30	13.05 (-4.87 to 30.97)	-0.99 (-18.55 to 16.56)
50th percentile of baseline RDQ (= 14)	70.24 ± 25.30	-4.26 (-16.51 to 7.98)	-0.89 (-13.16 to 11.28)
90th percentile of baseline RDQ (= 19)	75.50 ± 25.30	-21.58 (-39.56 to -3.59)*	-0.78 (-19.11 to 17.55)
Walking (m)††	419.33 ± 66.44	-7.80 (-29,60 to 13.99)	-22.83 (-44.72 to -0.93)*
Inversion fast walking (1/sec)	0.11 ± 0.02	-0.003 (-0.010 to 0.004)	0.001 (-0.006 to 0.008)
Inversion sit to stand (1/sec)	0.06 ± 0.02	-0.001 (-0.007 to 0.005)	-0.004 (-0.010 to 0.002)
Loaded forward reach (cm)††	58.70 ± 12.80	0.32 (-5.19 to 5.19)	0.21 (-5.30 to 5.71)
Stair climbing (stairs)	84.50 ± 22.31	-1.42 (-8.37 to 5.53)	-5.67 (-12.58 to 1.25)
Lifting (stages)††	4.87 ± 2.99	0.28 (-0.57 to 1.12)	-0.19 (-1.04 to 0.64)

† Values are the mean difference between this group and the CT. The CT score is the score at post-treatment.

The mean differences and confidence intervals (CIs) and corresponding p-values were estimated adjusting for age, gender, center of treatment, baseline score of outcome measure, duration of functional limitations.

†† Additional correction for relevant prognostic co-variates: RDQ – work status and TSK; Main complaints/Lifting – TSK; Current pain/PRI-T – work status; Global improvement/loaded forward reach – current pain; Walking – radiation and TSK.

$ Data presented for different strata for baseline RDQ, mean/SD for CT estimated by use of regression model. * p < 0.05.

APT = active physical treatment; CBT = cognitive-behavioural treatment; CT = combination treatment. RDQ = Roland Disability Questionnaire; PRI-T = Pain Rating Index Total score; BDI = Beck Depression Inventory.
